# Association of elevated cyclic GMP levels with hemodynamic changes in HFrEF patients treated with sacubitril/valsartan and vericiguat: a pilot study

**DOI:** 10.1016/j.ijcha.2025.101863

**Published:** 2026-01-07

**Authors:** Takumi Inoue, Hiroyuki Takahama, Hideaki Suzuki, Marina Arai, Nobuhiro Kikuchi, Taijyu Satoh, Nobuhiro Yaoita, Saori Yamamoto, Kotaro Nochioka, Makoto Nakano, Shunsuke Tatebe, Jun Takahashi, Naoto Minamino, Satoshi Yasuda

**Affiliations:** a Department of Cardiovascular Medicine, Tohoku University Graduate School of Medicine, Sendai, Japan; b Department of Cardiovascular Medicine, National Cerebral and Cardiovascular Ceter, Suita, Japan

**Keywords:** Heart failure, Cyclic GMP, Natriuretic peptide, Hemodynamic test

## Abstract

**Background:**

Patients with heart failure (HF) often present with a relative deficiency of cyclic guanosine monophosphate (cGMP) despite elevated B-type natriuretic peptide (BNP) levels. Sacubitril/valsartan and vericiguat target the cGMP pathway, but the relative contribution of cardiac versus systemic cGMP production remains uncertain. This study evaluated the association between cGMP changes and hemodynamic changes in patients with HF with reduced ejection fraction (HFrEF) receiving these agents.

**Methods:**

Fourteen symptomatic HFrEF patients (median age 65.0 [IQR: 56.0–72.3]years, EF 25.5 [24.0–33.3]%) and 20 control patients without HF (66.0 years, EF 66.5 %) were enrolled. Of the HFrEF patients, five received sacubitril/valsartan alone and nine received vericiguat (newly initiated or added to sacubitril/valsartan). All HFrEF patients underwent right heart catheterization before the treatment and two months after treatment. Blood samples were collected from the coronary sinus, arteries, and veins.

**Results:**

HFrEF patients showed higher coronary sinus cGMP levels compared with controls (15.8 ± 1.7 vs. 10.9 ± 1.2 nM, p < 0.05) but a markedly lower cGMP/BNP ratio (0.09 ± 0.02 vs. 1.71 ± 0.63, p < 0.05), suggesting a relative cGMP deficiency. After the therapy, the cGMP/BNP ratio significantly increased (0.278, p < 0.05). The change in coronary sinus cGMP correlated with improvement in cardiac index (r = 0.57, p = 0.039). cGMP levels rose consistently across all sampling sites, indicating a systemic augmentation of the cGMP pathway.

**Conclusion:**

Elevation of cGMP levels were associated with hemodynamic improvement in HFrEF patients treated with sacubitril/valsartan and vericiguat. These findings highlight the therapeutic relevance of cGMP pathway augmentation and provide mechanistic insights aligned with the known clinical effects of these agents in HFrEF.

## 1 Introduction

Cyclic guanosine monophosphate (cGMP) functions as a key intracellular second messenger. Its downstream effector, protein kinase G (PKG), modulates vascular tone, inhibits cardiac hypertrophy, and exerts cardioprotective effects [[1], [2], [3]]. Previous studies have reported a discrepancy between elevated cardiac stress and relatively low cGMP levels in heart failure (HF), a condition referred to as cGMP deficiency [[4], [5], [6]].

The cGMP–PKG pathway is activated through two major mechanisms: the natriuretic peptide (NP)–particulate guanylate cyclase (pGC) axis and the nitric oxide (NO)–soluble guanylate cyclase (sGC) axis. [7,8] Reduced NP signaling [4] and enhanced NP degradation [9,10] contribute to diminished cGMP generation. In addition, impaired processing of pro–B-type natriuretic peptide (proBNP), resulting in accumulation of biologically weak proBNP [11] and a decreased fraction of active BNP, further limits effective NP signaling. [6,12,13] This imbalance is reflected by a reduced cGMP-to-BNP (cGMP/BNP) ratio, [5,13] which may represent a biochemical marker of cGMP deficiency in HF [6].

Recent therapeutic advances have focused on restoring cGMP–PKG signaling in HF with reduced ejection fraction (HFrEF). Sacubitril/valsartan [14] and vericiguat [[15], [16], [17]] have been developed to enhance cGMP through stimulation of the pGC and sGC pathways, respectively. However, the extent to which cGMP augmentation translates into hemodynamic improvement in HFrEF remains unclear. Moreover, it is uncertain whether the cGMP-deficient state is primarily cardiac or systemic, and where the predominant therapeutic action of these agents occurs.

Therefore, the present study assessed cGMP levels in the artery, vein, and coronary sinus (CS) and examined whether changes in cGMP were associated with hemodynamic parameters in patients treated with sacubitril/valsartan or vericiguat. This analysis was conducted as a hypothesis-generating pilot study.

## 2 Methods

### 2.1 Study design

This prospective observational study included patients with HF admitted to Tohoku University Hospital between April 2022 and March 2025.

### 2.2 Study population

To compare biomarker levels and hemodynamic parameters between individuals with and without HF, the study included two groups: patients with HF (HF group) and those without HF (control group). HF was diagnosed according to the universal definition and classification of HF [18,19].

HF group: The HF group consisted of clinically stable patients with HF who were scheduled for right heart catheterization (RHC). The eligibility criteria were as follows: 1) hospital admission with HFrEF, defined as left ventricular ejection fraction (LVEF) ≤ 40 %, [19] and 2) provision of written informed consent. Exclusion criteria were as follows: 1) ongoing dialysis; 2) use of carperitide or intravenous nitrate within 24 h before blood sampling; 3) implantation of a cardiac resynchronization therapy (CRT) device, which precludes CS sampling; 4) estimated glomerular filtration rate (eGFR) < 30 mL/min/1.73 m^2^; and 5) prior use of both sacubitril/valsartan and vericiguat at enrollment. Patients were enrolled into two groups based on their scheduled treatment: (1) those newly initiating with sacubitril/valsartan and (2) those newly initiating with vericiguat. Among the 14 patients with HFrEF, five initiated sacubitril/valsartan and nine initiated vericiguat. Of the nine patients receiving vericiguat, four were already being treated with sacubitril/valsartan at the time of vericiguat initiation (Vericiguat add-on to ARNI), whereas the remaining five initiated vericiguat without prior sacubitril/valsartan use (Vericiguat without ARNI). The target doses for sacubitril/valsartan and vericiguat were the highest doses tolerated by the patients' clinical status in accordance with the guideline recommendation [19].

Control group (control): The control group consisted of patients without clinical HF or structural heart disease. Specifically, patients with paroxysmal atrial fibrillation (PAF) scheduled for radiofrequency catheter ablation were enrolled. All the participants provided written informed consent.

### 2.3 Blood sampling and hemodynamic measurements

Patients with HF underwent RHC during a clinically stable phase (baseline) and again two months after the initiation of sacubitril/valsartan or vericiguat therapy (post-med). This timeframe was chosen to allow for drug up-titration [14,15] and aligns with previous studies indicating that hemodynamic and biomarker changes typically emerge within this timeframe [5,16,20]. During RHC, blood samples were simultaneously collected from the CS using a catheter. Additional samples from the peripheral artery and vein were obtained through the radial or femoral artery and jugular vein sheaths, respectively, during RHC or radiofrequency catheter ablation procedures. Hemodynamic parameters, including pulmonary artery wedge pressure (PAWP), mean pulmonary artery pressure (PAP), right atrial pressure (RAP), and cardiac index, were measured at both time points using standard techniques during RHC.

### 2.4 Measurements of the biomarkers

Blood samples were collected in plastic tubes containing ethylenediaminetetraacetic acid (EDTA-2Na, 1.5 mg/mL) and aprotinin (500 kallikrein inhibitor units/mL). Plasma was separated by centrifugation and stored at –80 °C until analysis. As previously described [21], plasma levels of proBNP and total BNP (proBNP + mature (active) BNP) were measured using chemiluminescent enzyme immunoassays (CLEIAs), employing glycosylated proBNP (relative molecular mass 32,000 Da; HyTest Ltd., Turku, Finland) as a standard.[13] The percentage of proBNP was calculated as follows: (plasma proBNP/total BNP) × 100, as previously reported.[13] Plasma cGMP levels were quantified using a homogeneous time-resolved fluorescence (HTRF®) assay kit (Revvity Japan, Yokohama, Japan) [22] on 384-well plates (20 µL per well: sample 5 µL, assay diluent 5 µL, anti-cGMP Eu-cryptate 5 µL, cGMP-d2 reagent 5 µL). Plates were incubated for 60 min at room temperature and read on a PHERAstar FS plate reader (excitation 337 nm; emissions 620/665 nm); HTRF ratios (665/620 × 104) were converted to concentrations using a 4-parameter logistic calibration curve (0–500 nM). All measurements were analysed in duplicate; precision/acceptance followed the manufacturer’s specifications, and no phosphodiesterase inhibitors were added.

### 2.5 Echocardiography

Echocardiography was performed as a part of routine clinical care. The sonographers were blinded to the results of the biomarkers measured for the research. Echocardiographic findings were retrospectively reviewed from medical records. LV dimensions were measured according to the American Society of Echocardiography guidelines [23]. LVEF was assessed using the modified Simpson’s biplane method or the Teichholz method, as previously described [23].

### 2.6 Ethics

Written informed consent was obtained from all subjects. This study was approved by ethics committee of Tohoku University (2021-1-1077) and was conducted in accordance with the Declaration of Helsinki.

### 2.7 Statistical analysis

Continuous variables are presented as median and interquartile range (IQR). Categorical variables were compared using Fisher's exact test or the χ2 test, as appropriate. Wilcoxon’s rank-sum test was used to compare continuous variables between the two groups at baseline. Changes in hemodynamic parameters over time were assessed using one-way repeated-measures ANOVA. Associations between two continuous variables were evaluated using general linear regression analysis. All statistical tests were two-tailed, with a significance threshold of p < 0.05. Analyses were performed using R version 4.1.3 (R Foundation for Statistical Computing, Vienna, Austria; URL: https://www.R-project.org) and GraphPad Prism version 6.01 (GraphPad Software, San Diego, CA, USA).

## 3 Results

**Fig. 1:**
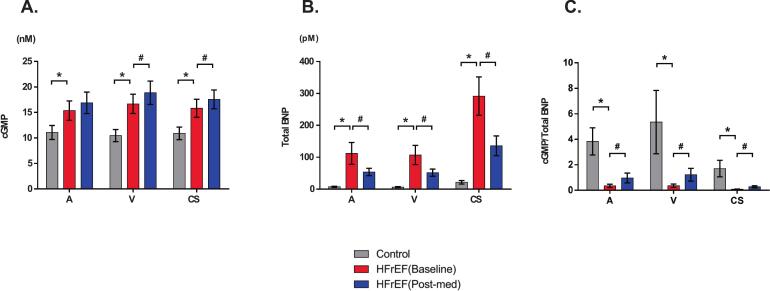
Plasma cGMP levels, total BNP levels, and cGMP/total BNP ratios in arterial, venous, and coronary sinus blood samples. A: cGMP levels in arterial (A), venous (V), and coronary sinus (CS) samples. B: Total BNP levels in each blood sample. C: cGMP/total BNP ratios in each sample. Gray, red, and blue bars represent the control group, HFrEF at Baseline, and HFrEF after treatment (Post-med), respectively. *: p<0.05 vs. control, #: p<0.05 vs. HFrEF (Baseline).

**Fig. 2:**
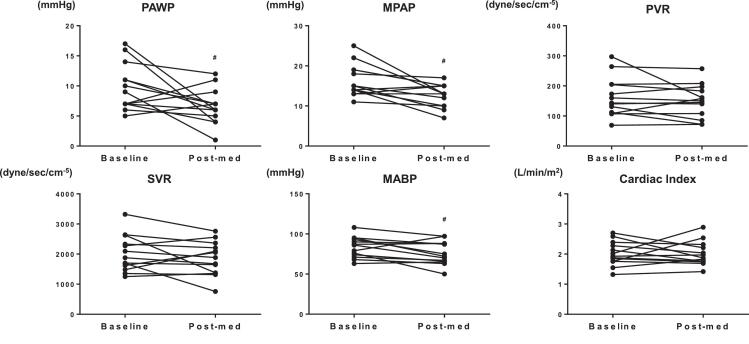
Changes in hemodynamic parameters assessed by right heart catheterization. Each panel shows the change in hemodynamic parameters between Baseline and after treatment (Post-med). PAWP, pulmonary artery wedge pressure; MPAP, mean pulmonary arterial pressure; PVR, pulmonary vascular resistance; SVR, systemic vascular resistance; MABP, mean arterial blood pressure. #: p < 0.05, vs. Baseline.

**Fig. 3:**
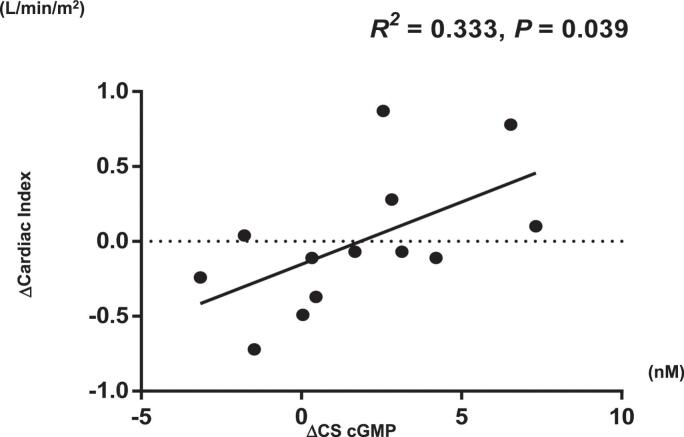
Correlation between changes in cGMP levels (ΔcGMP) and changes in cardiac index (ΔCardiac Index) in CS blood samples from Baseline to Post-med in patients with HFrEF. A: Correlation in all HFrEF patients. B: Correlation in patients with Baseline CS cGMP levels below the median value (14.6 nM). C: Correlation in patients with Baseline CS cGMP levels above the median value.

The baseline characteristics of the control and HF groups are summarized in Table 1. In the HF group, 85.7 % of patients were classified as NYHA class II, with a median LVEF of 25.5 % [IQR: 24.0, 33.3] and 42.9 % of patients had a history of HF hospitalization. In contrast, all patients in the control group were NYHA class I, with a median LVEF of 66.5 % [IQR: 60.8, 69.5]. The patient background of each sub-group at baseline were shown in Supplemental Table 1. The median doses of sacubitril/valsartan and vericiguat at the Postmed were 200 mg and 5.0 mg, respectively. Fig. 1 presents the plasma cGMP levels, plasma total BNP levels, and the cGMP/total BNP ratios in arterial, venous, and CS blood samples from both groups. Plasma levels of cGMP at baseline were comparable across blood sampling sites (artery, vein, and CS), whereas BNP levels in the CS were significantly higher than those at other sites (p < 0.05). Patients with HF showed significantly higher cGMP and total BNP levels at all sampling sites than the control group (Fig. 1, Fig. 1). In contrast, the cGMP/total BNP ratio was significantly lower in the HF group (p < 0.05; Fig. 1C). Following treatment with sacubitril/valsartan or vericiguat (see subgroup demographics in Supplementary Table 1), cGMP levels increased in both CS and venous samples (Fig. 1A), while total BNP levels declined across all sampling sites (Fig. 1B). No significant differences in cGMP levels [average: 16.9 ± 2.0 nM (artery), 18.9 ± 2.2 nM (vein) and 17.6 ± 1.8 nM (CS)] were observed among the various sampling sites following treatment (Fig. 1A). Consequently, the cGMP/total BNP ratio increased significantly after treatment compared to baseline (Fig. 1C) (Subgroup results for biomarker measurements are detailed in Supplementary Table 2). Hemodynamic data from the RHC are shown in Fig. 2. Regarding hemodynamics, mean PAWP and PAP decreased significantly after treatment (PAWP: 9.0 to 6.0 mmHg; PAP: 15 to 13 mmHg), whereas cardiac index remained unchanged (1.9 vs. 1.9 L/min/m^2^). The results of RHC in the sub-groups are shown in Supplemental Table 3. Fig. 3 shows the correlation between changes in CS cGMP levels (ΔcGMP) and changes in hemodynamic parameters of cardiac index. No significant associations were observed with other parameters. In addition, the correlation between ΔBNP and ΔcGMP was not statistically significant (Supplemental Fig. 1). Additionally, %proBNP in CS showed an inverse correlation with CS cGMP (r = −0.64, p = 0.014), suggesting that impaired proBNP processing may influence cGMP levels.

**Table 1:** Baseline Characteristics

	Control group (*n* = 20)	HF group (*n* = 14)	*P*-value
Age (years)	66.0 (64.5–70.3)	65.0 (56.0–72.5)	0.53
Female, n (%)	8 (40.0)	3 (21.4)	0.30
BMI (kg/m^2^)	22.9 (21.0–26.3)	23.9 (22.1–26.3)	0.73
Systolic BP (mmHg)	134 (127–142)	106 (96–112)	<0.001
Heart rate (/min)	74 (67–82)	71 (67–77)	0.54
**HF etiology**, n (%)			
Ischemic cardiomyopathy	0 (0)	1 (7.1)	0.41
Non ischemic cardiomyopathy	0 (0)	13 (92.9)	<0.001
**Clinical history**, n (%)			
Hypertension	14 (70.0)	4 (28.6)	0.035
Diabetes mellitus	0 (0)	1 (7.1)	0.41
Dyslipidemia	7 (35.0)	5 (35.7)	1
Paroxysmal atrial fibrillation	20 (100.0)	3 (21.4)	<0.001
Persistent atrial fibrillation	0 (0)	5 (35.7)	0.007
HF hospitalization	0 (0)	6 (42.9)	0.002
Myocardial infarction	0 (0)	1 (7.1)	0.41
Stroke	0 (0)	3 (21.4)	0.06
**NYHA class**, n (%)			
I	20 (100.0)	0 (0)	<0.001
II	0 (0)	12 (85.7)	<0.001
III-IV	0 (0)	2 (14.3)	0.16
**HF classification by LVEF**, n (%)			
HFrEF	0 (0)	14 (100.0)	<0.001
**Echocardiography**			
LVEDD, mm	46.5 (43.0–47.8)	59.0 (56.5–66.5)	<0.001
LAD, mm	34.0 (29.5–37.5)	38.5 (35.3–47.5)	0.014
LVEF, %	66.5 (60.8–69.5)	25.5 (24.0–33.3)	<0.001
**Laboratory data on admission**			
eGFR, mL/min/1.73m^2^	67.5 (58.5–80.3)	56.5 (47.3–66.8)	0.016
Total BNP, pg/mL	12.3 (9.1–33.8)	226.7(137.5–396.2)	<0.001
**Treatment**, n (%)			
Beta-blockers	6 (30.0)	13 (92.9)	<0.001
ACEi or ARB	8 (40.0)	9 (64.3)	0.30
ARNi	0 (0)	4 (28.6)	0.022

Aldosterone antagonists	0 (0)	12 (85.7)	<0.001

SGLT2i	0 (0)	11 (78.6)	<0.001
DOAC	20 (100.0)	8 (57.1)	0.002

Warfarin	0 (0)	2 (14.3)	0.16
Loop Diuretics	0 (0)	6 (42.9)	0.002

Pacemaker/ICD	0 (0)	1 (7.1)	0.41

Values are presented as median (interquartile range) or counts (percentages). HF, heart failure; HFrEF, heart failure with reduced ejection fraction; BMI, body mass index; BP, blood pressure; NYHA, New York Heart Association; LVEDD, left ventricular end-diastolic diameter; LAD, left atrial diameter; LVEF, left ventricular ejection fraction; eGFR, estimated glomerular filtration rate; BNP, B-type natriuretic peptide; ACEi, angiotensin-converting enzyme inhibitor; ARB, angiotensin II receptor blocker; ARNi, angiotensin receptor/neprilysin inhibitor; SGLT2i, sodium-glucose cotransporter-2 inhibitor; DOAC, direct oral anticoagulant; ICD, implantable cardioverter defibrillator.

## 4 Discussion

The major findings of this study were as follows: (1) although cGMP levels were elevated in patients with HF, the cGMP/BNP ratio was significantly lower than in controls; (2) both cGMP levels and the cGMP/BNP ratio were increased after treatment with sacubitril/valsartan or vericiguat; (3) a modest statistical association was observed between changes in CS cGMP levels and changes in cardiac index; and (4) baseline CS cGMP levels were inversely associated with %proBNP, suggesting a link between impaired proBNP processing and reduced cGMP activity in HFrEF; and (5) Although BNP levels were higher in the CS, post-treatment cGMP increases were not confined to the samples from the CS, with no significant gradients among sampling sites. These findings suggest that the elevated cGMP levels in patients treated with sacubitril/valsartan and vericiguat are associated with hemodynamic improvements, and that these effects are mainly mediated through systemic vascular mechanisms.

In a previous investigation conducted before the introduction of sacubitril/valsartan and vericiguat, circulating cGMP levels were observed to decline from the acute decompensated to the stabilized phase of HF after hemodynamic optimization [13]. This temporal pattern suggests that the transient elevation of cGMP during the acute phase represents compensatory activation of the NP–cGMP pathway in response to increased ventricular wall stress [24]. In contrast, the present study, which included patients with chronic and symptomatic HFrEF receiving contemporary medical therapy, demonstrated persistently higher circulating cGMP levels compared with controls. This may reflect sustained neurohormonal activation, pharmacologic augmentation of the cGMP pathway, or both. In addition, there were no significant relation of changes in cGMP levels with changes in BNP levels (Supplemental Fig. 1). This finding suggests that the observed increase in cGMP levels is not merely a passive result of the declines of BNP levels due to improvements of HF. Instead, this simultaneous increase in cGMP suggests the existence of direct pharmacological augmentation by the cGMP-modulating agents (sacubitril/valsartan and vericiguat) alongside the hemodynamic improvement. These findings collectively indicate that systemic cGMP activity remains upregulated in chronic HF and may serve as an integrated marker of ongoing cardiovascular stress or therapeutic engagement of cGMP-modulating interventions.

The cardioprotective effects of cGMP signaling are well established, encompassing vasodilation, inhibition of hypertrophy and fibrosis, enhancement of myocardial relaxation, and suppression of maladaptive neurohormonal activation, largely mediated through the cGMP–PKG axis [25,26]. The present study extends this concept by providing evidence that the therapeutic effects of cGMP-enhancing agents may primarily arise from systemic vascular rather than myocardial sources. The absence of a significant CS–arterial gradient in cGMP levels after treatment supports this interpretation, suggesting that the systemic vasculature is the predominant contributor to circulating cGMP in chronic HFrEF. These findings are compatible with predominantly systemic vascular effects, while not excluding potential myocardial actions, given that intracellular cGMP cannot be assessed in this study.

A notable finding was the significantly lower cGMP/BNP ratio in patients with chronic HFrEF compared with controls, despite elevated BNP concentrations. This discrepancy indicates impaired downstream activation of the NP–cGMP signaling cascade [6], potentially due to diminished NP bioactivity, enhanced peptide degradation, decreased receptor responsiveness, or disruption of intracellular transduction. The clinical implications of this impaired pathway are underscored by trials showing that pharmacologic augmentation of cGMP signaling with sacubitril/valsartan [14] or vericiguat [15] improves clinical outcomes, including reductions in cardiovascular mortality [27]. In the PARADIGM-HF trial, sacubitril/valsartan treatment increased the cGMP/BNP ratio relative to enalapril, and a higher ratio was associated with improved prognosis [5]. In alignment with these prior observations, the present study showed that cGMP levels and cGMP/BNP ratios were increased in patients treated with either sacubitril/valsartan or vericiguat, and that the degree of cGMP changes were modestly associated with improvements of cardiac index.

An inverse association between baseline CS cGMP levels and %proBNP was also identified, suggesting that impaired proBNP processing may attenuate activation of the NP–cGMP pathway in HFrEF. A higher proportion of circulating proBNP, indicative of inefficient cleavage of precursor peptides into biologically active BNP, has been reported in advanced HF [13]. This inefficient processing could contribute to a relative cGMP-deficient state despite elevated total BNP levels. Prior studies have suggested that this process may be influenced by O-linked glycosylation mediated by GalNAc-transferases, which are themselves regulated by microRNAs such as miR-30 [28]. Such mechanisms may partially explain interindividual differences in NP bioactivity and responsiveness to pharmacologic interventions. These observations emphasize the therapeutic rationale for agents that either prevent NP degradation (as with sacubitril/valsartan) or stimulate soluble guanylate cyclase directly (as with vericiguat), thereby compensating for upstream signaling impairments.

Although treatment with these cGMP-modulating therapies increased cGMP concentrations in the coronary sinus, the absence of a discernible arteriovenous or *trans*-cardiac gradient suggests that this rise primarily reflects systemic rather than cGMP generation in the heart. This finding indicates that the hemodynamic and clinical benefits of these agents are largely mediated through systemic augmentation of cGMP signaling rather than restoration of myocardial cGMP synthesis. Taken together with the VICTORIA trial [15] results showing a modest reduction in mortality and morbidity with vericiguat in patients with chronic HFrEF, the present findings offer mechanistic insights that align with the clinical effects reported in these high-risk cohorts. This clarification emphasizes that our physiological observations provide supportive mechanistic context, while acknowledging that the overall clinical efficacy of sGC stimulation remains limited and should be interpreted cautiously. While CS sampling is not feasible for routine clinical practice, it provides unique pathophysiological insight into compartmentalized cGMP regulation and underscores the need for non-invasive strategies to assess cGMP activity in future studies.

## 5 Limitations

This study had several limitations. First, the study was conducted at a single center with a relatively small patient cohort, which may have limited the generalizability of the findings. Although statistical significance was observed in the linear regression analyses (Fig. 3), the findings possess inherent fragility due to the small sample size and limited statistical power. We acknowledged this study as an exploratory pilot investigation, and ideally, further large-scale, multi-center studies are necessary to validate the observed compartmentalized cGMP changes and their link to cardiac function. Second, CS sampling could not be performed in some patients because of technical difficulties, which resulted in their exclusion. Third, given the strong guideline recommendations for sacubitril/valsartan and vericiguat in HFrEF, it is not ethically feasible to delay the initiation of these treatments for study purposes. Patients in this study were also receiving other optimized HF therapies, which may have contributed to the observed hemodynamic and biomarker changes. Therefore, the findings cannot be ascribed specifically to cGMP-modulating agents. Patients with PAF scheduled for radiofrequency catheter ablation were included in the control group. PAF patients are not healthy controls and that absolute cGMP/BNP levels should be interpreted with caution. Although this may be considered a limitation, these patients were selected to ensure the safe and ethical collection of CS blood samples during the procedure. Furthermore, because each treatment subgroup included only 4–5 patients, statistical comparisons of RHC parameters or biomarkers between sacubitril/valsartan and vericiguat exposure groups were not conducted. The requirement for successful CS sampling for inclusion introduced a potential selection bias, as patients with difficult venous access or specific anatomical variants were necessarily excluded (n = 4). Lastly, the selection of a two-month follow-up period may also be a limitation, as the cGMP/BNP ratio remained significantly lower in patients with HFrEF than in the controls at this time point, and a longer follow-up may be necessary to capture more complete pathway recovery.

## 6 Conclusion

This study identified a relative cGMP deficiency in patients with HFrEF, and demonstrated association of elevated cGMP levels with hemodynamic changes in HFrEF patients treated with sacubitril/valsartan and vericiguat. These findings offer preliminary insights into cGMP-related hemodynamic patterns in this treated cohort and are consistent with known clinical effects of cGMP-modulating therapy in HFrEF.

## 7 Sources of funding

This study was supported, in part, by Grants-in Aid from the Ministry of Health, Labour, and Welfare and the Ministry of Education, Culture, Sports, Science, and Technology.

8 Ethical StatementN/A.

## CRediT authorship contribution statement

**Takumi Inoue:** Writing – original draft, Methodology, Investigation, Formal analysis, Data curation. **Hiroyuki Takahama:** Writing – original draft, Supervision, Project administration, Methodology, Investigation, Conceptualization. **Hideaki Suzuki:** Writing – review & editing, Validation, Investigation, Data curation. **Marina Arai:** Methodology, Investigation. **Nobuhiro Kikuchi:** Methodology, Investigation, Data curation. **Taijyu Satoh:** Validation, Methodology, Investigation, Data curation. **Nobuhiro Yaoita:** Methodology, Investigation, Data curation, Conceptualization. **Saori Yamamoto:** Methodology, Investigation, Data curation. **Kotaro Nochioka:** Methodology, Investigation, Formal analysis, Data curation. **Makoto Nakano:** Project administration, Methodology, Investigation, Data curation. **Shunsuke Tatebe:** Methodology, Investigation, Data curation. **Jun Takahashi:** Project administration, Methodology, Investigation, Conceptualization. **Naoto Minamino:** Validation, Methodology, Investigation, Conceptualization. **Satoshi Yasuda:** Writing – review & editing, Validation, Supervision, Conceptualization.

## Declaration of competing interest

The authors declare the following financial interests/personal relationships which may be considered as potential competing interests: T Inoue, H Suzuki, M Arai, N Kikuchi, T Satoh, N Yaoita, S Yamamoto, S Tatebe, J Takahashi, and N Minamino have nothing to disclose. H Takahama reports lecture fee from Novartis Pharmaceuticals and Bayer. K Nochioka reports lecture fee from Novartis Pharmaceuticals and Bayer. M Nakano reports lecture fee from Bayer. S Yasuda reports grant from Novartis Pharmaceuticals.
