# Supplementary figures and images for: Association of elevated cyclic GMP levels with hemodynamic changes in HFrEF patients treated with sacubitril/valsartan and vericiguat: a pilot study
Source: Int J Cardiol Heart Vasc. 2026 Jan 7;62:101863. doi: 10.1016/j.ijcha.2025.101863 (PMC13153138; doi:10.1016/j.ijcha.2025.101863)

## Slide 1
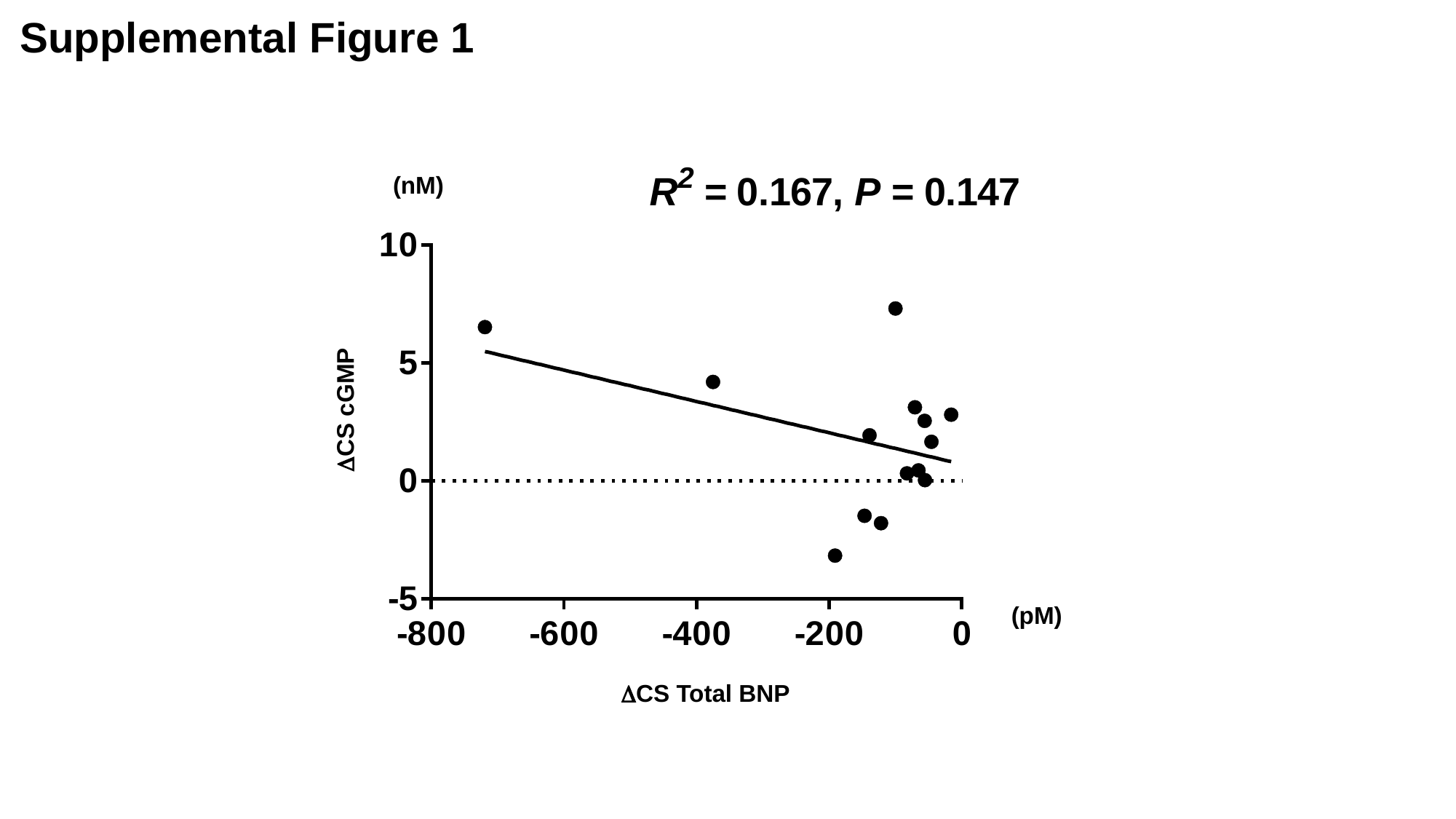

Supplemental Figure 1
(nM)
DCS cGMP
(pM)
DCS Total BNP

Supplement: Supplementary Data 1 [file mmc1.pptx]
