# Supplementary material for: Association of elevated cyclic GMP levels with hemodynamic changes in HFrEF patients treated with sacubitril/valsartan and vericiguat: a pilot study
Source: Int J Cardiol Heart Vasc. 2026 Jan 7;62:101863. doi: 10.1016/j.ijcha.2025.101863 (PMC13153138; doi:10.1016/j.ijcha.2025.101863)
Supplement: Supplementary Data 2 [file mmc2.docx]

**Supplemental Table 1. Baseline Characteristics According to Sacubitril/valsartan and Vericiguat Treatment Status.**

|  | | ARNI only  (*n* = 5) | Vericiguat Add-on to ARNi  (*n* = 4) | Vericiguat without ARNi  (*n* = 5) |
| --- | --- | --- | --- | --- |
| Age (years) | | 54.0 (47.0-65.0) | 65.0 (57.8-69.0) | 71.0 (65.0-73.0) |
| Female, n (%) | | 2 (40.0) | 0 (0) | 1 (20.0) |
| BMI (kg/m^2^) | | 22.1 (19.9-22.3) | 25.6 (24.7-25.9) | 26.5 (23.5-27.7) |
| Systolic BP (mmHg) | | 112 (111-118) | 102 (96-108) | 105 (93-106) |
| Heart rate (/min) | | 71 (71-78) | 72 (70-77) | 66 (54-68) |
| **Echocardiography** | |  |  |  |
|  | LVEDD, mm | 58.0 (56.0-59.0) | 63.0 (58.3-69.8) | 64.0 (59.0-65.0) |
|  | LAD, mm | 34.0 (32.0-40.0) | 49.5 (43.5-55.0) | 37.0 (36.0-48.0) |
|  | LVEF, % | 26.0 (25.0-34.0) | 25.0 (22.0-28.8) | 24.0 (24.0-31.0) |
| **Laboratory data on admission** | |  |  |  |
|  | eGFR, mL/min/1.73m^2^ | 63.0 (62.0-75.0) | 54.0 (51.0-59.0) | 44.0 (36.0-47.0) |
|  | Total BNP, pg/mL | 104.4  (27.7-134.4) | 300.6  (253.5-343.5) | 423.6  (205.1-589.0) |
| **Treatment at Baseline**, n (%) | |  |  |  |
|  | Beta-blockers | 5 (100.0) | 4 (100.0) | 5 (100.0) |
|  | ACEi or ARB | 4 (80.0) | 0 (0) | 5 (100.0) |
|  | ARNi | 0 (0) | 4 (100.0) | 0 (0) |
|  | Aldosterone antagonists | 4 (80.0) | 4 (100.0) | 5 (100.0) |
|  | SGLT2i | 5 (100.0) | 4 (100.0) | 5 (100.0) |

**ARNI only**: Sacubitril/valsartan was newly initiated in patients who had not received prior sacubitril/valsartan or vericiguat therapy. **Vericiguat Add-on to ARNI**

: Vericiguat was introduced in patients who had been previously treated with sacubitril/valsartan. **Vericiguat without ARNI**: Vericiguat was initiated in patients who had not received prior sacubitril/valsartan treatment.

Values are presented as median (interquartile range) or counts (percentages). BMI, body mass index; BP, blood pressure; LVEDD, left ventricular end-diastolic diameter; LAD, left atrial diameter; LVEF, left ventricular ejection fraction; eGFR, estimated glomerular filtration rate; BNP, B-type natriuretic peptide; ACEi, angiotensin-converting enzyme inhibitor; ARB, angiotensin II receptor blocker; ARNi, angiotensin receptor/neprilysin inhibitor; SGLT2i, sodium-glucose cotransporter-2 inhibitor.
