# Supplementary material for: Association of elevated cyclic GMP levels with hemodynamic changes in HFrEF patients treated with sacubitril/valsartan and vericiguat: a pilot study
Source: Int J Cardiol Heart Vasc. 2026 Jan 7;62:101863. doi: 10.1016/j.ijcha.2025.101863 (PMC13153138; doi:10.1016/j.ijcha.2025.101863)
Supplement: Supplementary Data 3 [file mmc3.docx]

**Supplementary Table 2. Biomarker Changes in HFrEF patients treated with Sacubitril/valsartan and Vericiguat.**

|  | | ARNi Only  (*n* = 5) | | vericiguat add-on to ARNI  (*n* = 4) | | vericiguat without ARNI  (*n* = 5) | |
| --- | --- | --- | --- | --- | --- | --- | --- |
| **Biomarkers in CS blood** | | Baseline | Post-med | Baseline | Post-med | Baseline | Post-med |
|  | Total BNP (pM) | 128.6  (99.4-157.1) | 34.4  (27.3-58.3) | 409.1  (242.9-581.8) | 193.5  (115.1-301.6) | 299.5  (166.4-354.4) | 110.8  (106.4-160.8) |
|  | cGMP (nM) | 16.1 ± 3.0 | 17.1 ± 2.9 | 18.0 ± 3.6 | 17.9 ± 3.6 | 13.8 ± 2.0 | 17.8 ± 2.8 |
|  | cGMP/Total BNP | 0.100  (0.084-0.193) | 0.479  (0.196-0.849) | 0.035  (0.032-0.054) | 0.080  (0.053-0.173) | 0.063  (0.035-0.074) | 0.134  (0.117-0.135) |

**ARNI only**: Sacubitril/valsartan was newly initiated in patients who had not received prior sacubitril/valsartan or vericiguat therapy. **vericiguat add-on to ARNI**: Vericiguat was introduced in patients who had been previously treated with sacubitril/valsartan. **vericiguat without ARNI**: Vericiguat was initiated in patients who had not received prior sacubitril/valsartan treatment.

Values are presented as median (interquartile range) or mean ± SEM. CS, coronary sinus; BNP, B-type natriuretic peptide; cGMP, cyclic guanosine monophosphate.
