# Supplementary material for: Association of elevated cyclic GMP levels with hemodynamic changes in HFrEF patients treated with sacubitril/valsartan and vericiguat: a pilot study
Source: Int J Cardiol Heart Vasc. 2026 Jan 7;62:101863. doi: 10.1016/j.ijcha.2025.101863 (PMC13153138; doi:10.1016/j.ijcha.2025.101863)
Supplement: Supplementary Data 4 [file mmc4.docx]

**Supplemental Table 3. Hemodynamic Changes in HFrEF patients treated with Sacubitril/valsartan and Vericiguat Treatment Status.**

|  | | ARNI only  (*n* = 5) | | vericiguat add-on to ARNI (*n* = 4) | | vericiguat without ARNI  (*n* = 5) | |
| --- | --- | --- | --- | --- | --- | --- | --- |
| **Hemodynamics** | | Baseline | Post-med | Baseline | Post-med | Baseline | Post-med |
|  | PAWP (mmHg) | 9.0  (7.0-10.0) | 5.0  (4.0-6.0) | 12.5  (10.0-14.5) | 9.0  (6.3-11.3) | 7.0  (7.0-10.0) | 6.5  (6.0-7.5) |
|  | MPAP (mmHg) | 14.0  (14.0-15.0) | 10.0  (9.0-12.0) | 19.0  (17.5-20.5) | 15.0  (14.5-15.0) | 16.0  (14.0-18.0) | 14.0  (12.3-15.5) |
|  | PVR (dyne/sec/cm^-5^) | 108  (107-140) | 145  (108-158) | 152  (126-181) | 141  (82-200) | 163  (160-204) | 156  (148-167) |
|  | SVR (dyne/sec/cm^-5^) | 1700  (1621-2276) | 2096  (2054-2561) | 1616  (1330-1991) | 1518  (1349-1810) | 2095  (1796-2629) | 1632  (1222-2006) |
|  | MABP (mmHg) | 87  (86-90) | 88  (88-97) | 81  (67-94) | 70  (65-78) | 74  (71-76) | 67  (62-68) |
|  | Cardiac Index (L/min/m^2^) | 2.4  (2.1-2.6) | 1.9  (1.8-2.2) | 1.9  (1.9-2.0) | 1.9  (1.8-2.0) | 1.8  (1.5-1.8) | 2.1  (1.6-2.6) |

**ARNI initiation**: Sacubitril/valsartan was newly initiated in patients who had not received prior sacubitril/valsartan or vericiguat therapy. **vericiguat add-on to ARNI**: Vericiguat was introduced in patients who had been previously treated with sacubitril/valsartan. vericiguat without ARNI: Vericiguat was initiated in patients who had not received prior sacubitril/valsartan treatment.

Values are presented as median (interquartile range). PAWP, pulmonary artery wedge pressure; MPAP, mean pulmonary artery pressure; PVR, pulmonary vascular resistance; SVR, systemic vascular resistance; MABP, mean arterial blood pressure.
